# A small molecule compound IMB-LA inhibits HIV-1 infection by preventing viral Vpu from antagonizing the host restriction factor BST-2

**DOI:** 10.1038/srep18499

**Published:** 2015-12-16

**Authors:** Zeyun Mi, Jiwei Ding, Quan Zhang, Jianyuan Zhao, Ling Ma, Haisheng Yu, Zhenlong Liu, Guangzhi Shan, Xiaoyu Li, Jinming Zhou, Tao Wei, Liguo Zhang, Fei Guo, Chen Liang, Shan Cen

**Affiliations:** 1 Institute of Medicinal Biotechnology, Chinese Academy of Medical Sciences and Peking Union Medical School, Beijing 100050, China; 2 Beijing Union University, Beijing 100101, China; 3 Lady Davis Institute-Jewish General Hospital, McGill University, Montreal, Quebec, Canada; 4 Institute of Pathogen Biology, Chinese Academy of Medical Science, Beijing, China; 5 Department of Biochemistry and Molecular Biology, Tianjin Medical University, Tianjin, China; 6 Institute of Biophysics, Chinese Academy of Sciences, Beijing, China

## Abstract

Human BST-2 inhibits HIV-1 replication by tethering nascent virions to the cell surface. HIV-1 codes Vpu that counteracts BST-2 by down-regulating this restriction factor from the cell surface. This important function makes Vpu a potential therapeutic target. Yet, no agents have been reported to block Vpu from antagonizing BST-2. In this study, we report a small molecule compound IMB-LA that abrogates the function of Vpu and thereby strongly suppresses HIV-1 replication by sensitizing the virus to BST-2 restriction. Further studies revealed that IMB-LA specifically inhibits Vpu-mediated degradation of BST-2 and restores the expression of BST-2 at the cell surface. Although IMB-LA does not prevent Vpu from interacting with BST-2 or β-TrCP2-containing ubiquitin E3 ligase, sorting of BST-2 into lysosomes in Vpu-expressing cells is blocked by IMB-LA. Most importantly, HIV-1 release and infection is inhibited by IMB-LA only in BST-2-expressing cells. In summary, results herein demonstrated that IMB-LA could specifically inhibit the degradation of BST-2 induced by Vpu, and impair HIV-1 replication in a BST-2 dependent manner, suggesting the feasibility of utilizing small molecule compounds to disable the antagonist function of Vpu and thereby expose HIV-1 to the restriction by BST-2.

Since its first practice in clinics thirty years ago, highly active antiretroviral therapy (HAART) has greatly improved the life expectancy of AIDS patients. At the core of HAART is a repertoire of potent HIV inhibitors. The inevitable development of drug resistance and unavailability of effective vaccines necessitate continuous discovery of new drugs to renew the existing arsenal of HIV inhibitors. Despite the existence of many targets in HIV life cycle that can be pharmacologically intervened, only five targets are blocked by the currently prescribed HIV inhibitors, these include reverse transcriptase, integrase, protease, envelop glycoprotein and the co-receptor CCR5. With the discovery of potent host restriction factors and the appreciation of the role of innate immunity in controlling HIV infection, more and more attention is paid to develop strategies to enhance the activities of host restriction factors as one means to battle HIV infection^1^. Our group and others have previously developed small molecule compounds that are able to block HIV-1 Vif protein from antagonizing host restriction factor called APOBEC3G and thereby expose HIV-1 to APOBEC3G inhibition^2^,^3^,^4^.

One recently discovered host restriction factor is BST-2 (also called tetherin, CD317 or HM1.24) that acts by blocking the release of diverse enveloped viruses^5^,^6^,^7^,^8^,^9^. BST-2 is mainly expressed in mature B cells and plasmacytoid dendritic cells, but can be induced in many other cell types by type-1 interferon (IFN)^10^,^11^,^12^. Human BST-2 consists of 180 amino acids and shows a type 2 membrane topology with a N-terminal cytoplasmic (CT) domain, followed by a transmembrane (TM) domain, a large extracellular domain containing two possible N-glycosylation sites, and a C-terminal glycophosphatidylinositol (GPI) anchor that serves as a second membrane binding site^5^,^9^,^13^. BST-2 is able to retain virus particles at the cell surface by physically linking the viral and plasma membranes, thereby impairing viral replication^9^,^14^. In addition, the fact that BST-2 can be incorporated into HIV-1 particles and can decrease the infectivity of the progeny viruses suggests another possible mechanism underlying the anti-HIV activity of BST-2^15^,^16^.

As a countermeasure, HIV-1 accessory protein viral protein U (Vpu) is able to interact with BST-2 and overcome its antiviral activity^9^,^17^,^18^,^19^. The early studies showed that Vpu contributes to down-regulation of the CD4 receptor. Vpu is able to degrade newly synthesized CD4 molecules that bind to viral envelope glycoproteins (Env) in the endoplasmic reticulum (ER) through the ubiquitin-proteasome pathway^20^,^21^,^22^. Recently, Vpu was found to promote HIV-1 particles release by suppressing the activity of human BST-2. A series of studies suggest that Vpu targets BST-2 to the trans-Golgi network (TGN) or to lysosomes/proteasomes for degradation via a β-TrCP2-dependent mechanism. In doing so, Vpu removes BST-2 from the cell surface and consequently enhances the release of the progeny viruses^17^,^18^,^19^,^23^,^24^,^25^,^26^. Vpu interacts with BST-2 via the trans-membrane domains (TMDs)^26^,^27^,^28^,^29^,^30^. Vpu utilizes the 51-DpSGxxpS-56 motif to interact with β-TrCP2 that is a substrate adaptor of an SCF ubiquitin E3 ligase complex^17^,^18^. Vpu thus recruits the E3 ligase complex to ubiquitinate BST-2^17^,^19^,^31^.

Theoretically, blocking the antagonist function of Vpu should expose HIV-1 to the potent restriction by BST-2 and therefore constitutes a new strategy to treat HIV-1 infection. Indeed, several attempts have been made to discover small molecular compounds targeting HIV-1 Vpu. BIT225, an amiloride analogues, was identified as a late-phase inhibitor targeting Vpu, which significantly inhibits HIV-1 release from both acutely and chronically infected macrophages^32^. However, subsequent work showed that BIT225 does not exert its antiviral function by inhibiting Vpu-mediated BST-2 down-regulation, rather by specifically targeting the viroporin function of Vpu^33^. In this work, we have screened a compound library for Vpu inhibitors utilizing a cell-based high throughput assay to monitor Vpu-mediated down-regulation of BST-2 from the surface of HeLa cells. The results revealed one leading compound IMB-LA that restored cell-surface BST-2 in the presence of Vpu. Importantly, IMB-LA inhibited the release and replication of wild type HIV-1 in BST-2-expressing cells. This work provides proof-of-concept evidence supporting novel strategies for anti-HIV drugs development.

## Results

### IMB-LA restores the level of cell surface BST-2 in a Vpu-dependent manner

**Figure 1:**
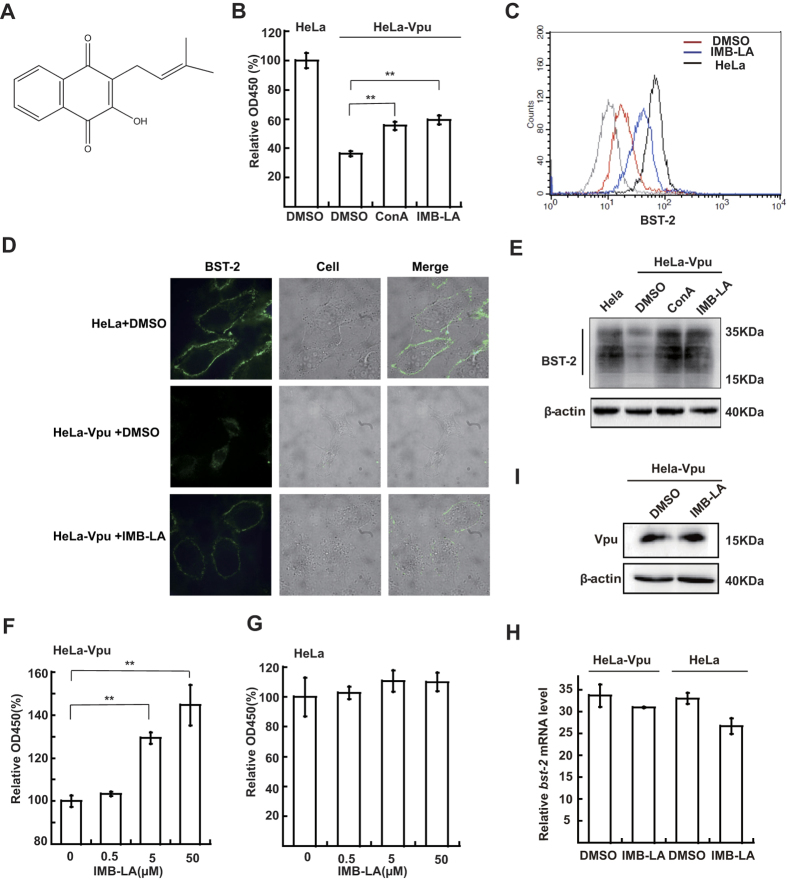
IMB-LA prevents Vpu from downregulating BST-2 from the cell surface. (**A**) The structure of IMB-LA. (**B**) HeLa-Vpu cells were treated with DMSO, 50 nM ConA and 5 μM IMB-LA for 24 h. Cell-surface BST-2 was determined by Cell-ELISA. (**C**) HeLa-Vpu cells were treated with DMSO, 5 μM IMB-LA for 24 h. The cells were immunostained for cell-surface BST-2 using anti-BST-2 antibody and FITC-conjugated goat-anti-rabbit secondary antibody, followed by flow cytometry. HeLa-Vpu cells were also stained with a control antibody and the signals serve as a control as represented by the gray line. BST-2 levels in HeLa-Vpu cells treated with DMSO and IMB-LA are indicated by the red line and blue line, respectively, the black line represents level of cell surface BST-2 in HeLa cells. (**D**) HeLa and HeLa-Vpu Cells were treated with DMSO or 5 μM IMB-LA. 24h later, cells were incubated with mouse anti-BST-2 antibodies and FITC-conjugated secondary antibodies. Images were recorded using a spinning disc confocal microscope. Signals of BST-2 are pseudocolored as Green. (**E**) Western blot analysis for BST-2 protein in HeLa-Vpu cells treated with DMSO, 50 nM ConA and 5 μM IMB-LA. BST-2 and β-actin expression were detected using an anti-BST-2 rabbit antibody (top panel) and anti-β-actin antibody (bottom panel). (**F**) HeLa-Vpu cells were treated with various concentrations of IMB-LA for 24 h and cell surface BST-2 was determined using Cell-ELISA. (**G**) HeLa cells were treated with different concentrations of IMB-LA for 24 h and cell surface BST-2 was determined using Cell-ELISA. (**H**) HeLa and HeLa-Vpu cells were treated with DMSO or 5 μM IMB-LA for 24 h. The amount of *bst*-2 mRNA was measured by real time RT-PCR and normalized with that of *gapdh* mRNA. (**I**) HeLa-Vpu cells were treated with DMSO and IMB-LA for 24 h. Cell lysates were examined by Western blotting using anti-Vpu and anti-β-actin antibodies. Each bar represents the mean and standard deviation of at least three independent experiments. **denotes *P* < 0.01. Full-length blots/gels are presented in Supplementary data.

To identify small molecules that inhibit Vpu-mediated degradation of BST-2, we previously developed a high-throughput screen (HTS) Cell-ELISA assay using a HeLa cell line that stably expresses Vpu (called HeLa-Vpu cell)^34^. Using this assay, we screened a compound library and identified one hit named IMB-LA that is known as lapachol (Fig. 1A). IMB-LA increased cell-surface BST-2 by approximate 50% in HeLa-Vpu cells (Fig. 1B and C). As a positive control, the lysosome inhibitor concanamycin A (ConA) also increased BST-2 expression in HeLa-Vpu cells through blocking Vpu-mediated lysosome degradation of BST-2 (Fig. 1B). To further validate the result from the ELISA and FACS assays, cell surface BST-2 was assessed by immune-fluorescence microscopy, and the result showed that BST-2 was increased at the cell surface in the presence of IMB-LA (Fig. 1D). Similarly, IMB-LA is capable of restoring the cellular content of BST-2 as ConA does (Fig. 1E). Further experiments showed dose-dependent up-regulation of cell-surface BST-2 by IMB-LA in HeLa-Vpu cells, but not in control HeLa cells that do not express Vpu (Fig. 1F and G), which suggests that IMB-LA restores BST-2 expression in a Vpu-dependent manner. IMB-LA barely affected the transcription of BST-2, as shown by the result of real-time RT-PCR that measured BST-2 mRNA (Fig. 1H). In order to examine whether IMB-LA increases BST-2 level by diminishing Vpu expression, level of Vpu was measured by Western blot and no effect by IMB-LA was observed (Fig. 1I). Taken together, these results suggest that IMB-LA increases cell-surface BST-2 by targeting Vpu-mediated BST-2 degradation.

### IMB-LA inhibits Vpu-mediated BST-2 down-regulation in a highly specific manner

**Figure 2:**
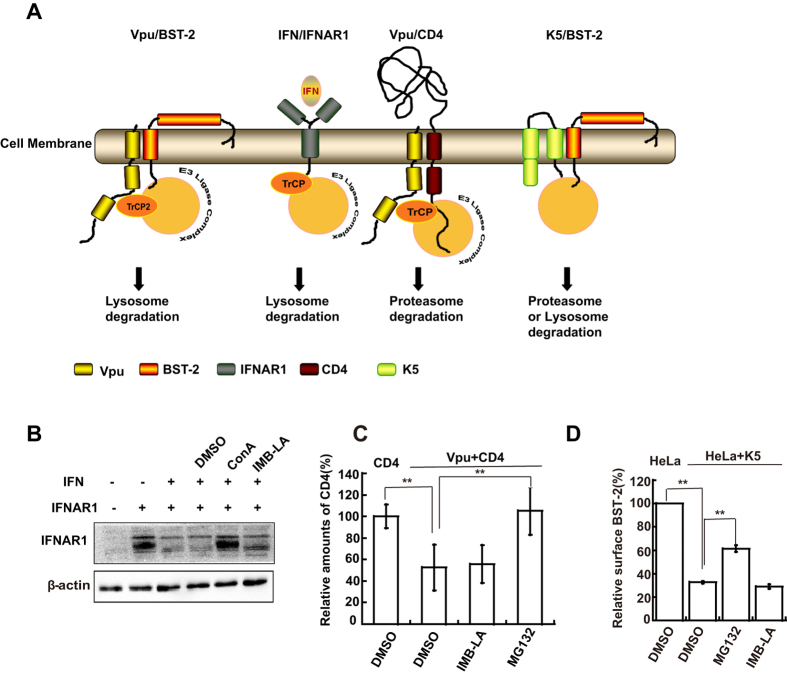
IMB-LA specifically inhibits Vpu-mediated degradation of BST-2. (**A**) Schematic illustration of downregulation of BST-2 and CD4 by Vpu. The downregulation of IFNAR1 upon treated with IFN-α, and the downregulation of BST-2 by K5 are also shown. (**B**) HeLa cells were transfected with pcDNA3.1-IFNAR1-FLAG or empty vector. 40 h later, cells were treated with different agents at the indicated concentration. IFN-α: 1000 U/ml; cycloheximide: 20 μg/ml; ConA: 50 nM; IMB-LA: 5 μM. Western blot was performed with anti-FLAG and anti-β-actin antibodies. (**C**) HEK293T cells were co-transfected with plasmids coding for human CD4 and HIV-1 Vpu. Cells were treated with DMSO and 5 μM IMB-LA. Then cells surface CD4 was stained and analyzed by flow cytometry. (**D**) HeLa cells were transfected with 500 ng pcDNA3.1-K5-HA per well in a 6-well plate. 24 h later, cells were treated with DMSO, MG132 and 5 μM IMB-LA for another 24 h. Then cells surface BST-2 was stained and analyzed by flow cytometry. Full-length blots/gels are presented in Supplementary data.

We next investigated how specifically IMB-LA interferes with the Vpu/BST-2 pathway. To this end, we first tested whether IMB-LA exerts a general effect on the lysosome degradation pathway which is involved in Vpu-mediated BST-2 degradation. We thus chose to measure the effect of IMB-LA on down-regulation of interferon-α receptor 1 (IFNAR1) by interferon α (IFNα), which occurs through ubiquitination of IFNAR1 by the Skp1-Cullin1- HOS-Roc1 (SCFHOS) ubiquitin E3 ligase and subsequent degradation via the lysosomal pathway^35^. HeLa cells were transfected with pcDNA3.1-IFNAR1-FLAG, then treated with IFNα. Either IMB-LA or ConA was added at the indicated concentrations. As shown in Fig. 2B, only ConA, not IMB-LA, was able to inhibit the down-regulation of IFNAR1 in the presence of IFNα. These results suggest that IMB-LA does not exert a general impact on the lysosome-dependent protein degradation.

We next tested whether IMB-LA affects Vpu-mediated down-regulation of CD4. It is known that that Vpu induces proteasome-dependent degradation of CD4 by recruiting to the multi-subunit SCF/β-TrCP (1 or 2) ubiquitin E3 ligase (Fig. 2A)^21^,^20^,^36^,^22^. As opposed to proteasome inhibitor MG132 that markedly increased cell-surface CD4 in the presence of Vpu, no such effect was detected for IMB-LA (Fig. 2C), which further supports the specificity of IMB-LA to rescue Vpu-mediated down-modulation of BST-2 but not other cellular proteins such as CD4.

Finally, we tested the effect of IMB-LA on another viral antagonist of BST-2, called K5 (also known as MIR2) of Kaposi’s sarcoma-associated herpesvirus (KSHV)^37^. K5 belongs to membrane-associated RING-CH (MARCH) protein that mediates ubiquitination of the cytoplasmic portion of transmembrane proteins^38^,^39^. K5 counteracts BST-2 by ubiquitinating BST-2 and targeting it for lysosomal degradation^37^,^39^. To determine whether IMB-LA inhibits K5-mediated down-regulation of BST-2, we transfected HeLa cells with pcDNA3.1-K5-HA, a plasmid that expresses K5, and then treated cells with IMB-LA. A significant down-regulation of BST-2 by K5 protein was observed. Yet, unlike MG132, IMB-LA failed to rescue this reduction of BST-2 in the presence of K5 (Fig. 2D). Taken together, these results demonstrate a high specificity of IMB-LA in inhibiting the down-regulation of BST-2 by Vpu.

### IMB-LA does not affect formation of the BST-2/Vpu/β-TrCP2 complex

**Figure 3:**
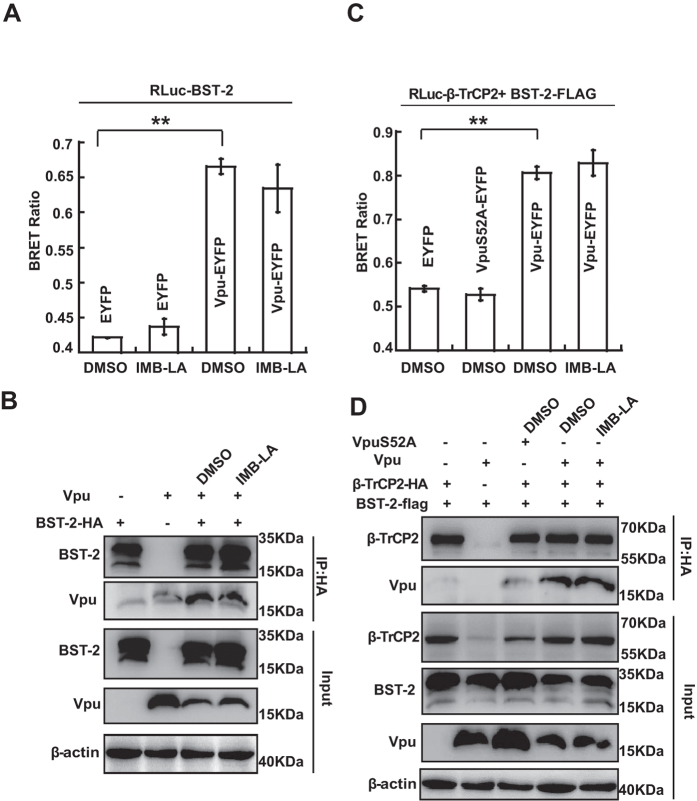
Effect of IMB-LA on the interactions of Vpu with BST-2 and β-TrCP2. (**A**) HEK293T cells were co-transfected with pEYFP-N1-Vpu and pRluc-C3-BST-2 at the ratio of 1:1. 24 h post-transfection, cells were treated with DMSO or 5 μM IMB-LA for 24 h. BRET ratio was measured as described in Methods. (**B**) HEK293T cells were co-transfected with the pcDNA3.1-Vpu and pcDNA3.1-BST-2-HA at the ratio of 1:1. 24 h later, cells were treated with the agents as described in Fig. 3A. Lysates were immunoprecipitated with rabbit anti-HA antibody and proteinA-Agrose followed by immunoblotted with anti-BST-2-HA and anti-Vpu antibodies. The pre-IP lysates represent 1% of the IP input and were also immunoblotted for β-actin as a loading control. (**C**) HEK293T cells were co-transfected with pEYFP-N1-Vpu, pRluc-C3-β-TrCP2-HA and pcDNA3.1-BST-2-FLAG (1:1:1). pEYFP-N1-VpuS52A was used as a negative control for this assay. 24 h post-transfection, cells were treated with DMSO and 5 μM IMB-LA for 24 h. BRET ratio was measured as described in Methods. (**D**) HEK293T cells were co-transfected with pcDNA3.1-Vpu, pcDNA3.1-β-TrCP2-HA and pcDNA3.1-BST-2 -FLAG (1:1:1) and treated with the agents as described in Fig. 3C. Lysates were immunoprecipitated with rabbit anti-HA antibody and proteinA-Agrose followed by immunoblotted with anti-β-TrCP2-HA and anti-Vpu antibodies. The pre-IP lysates represented 1% of the IP input and were also immunoblotted for β-actin as a loading control. Each bar represents the mean and standard deviation of at least three independent experiments. **denotes *P* < 0.01. Full-length blots/gels are presented in Supplementary data.

It is known that Vpu directly interacts with BST-2 and β-TrCP2, and that these two interactions are both required for Vpu to down-regulate BST-2^19^,^40^. We therefore asked whether IMB-LA overcomes the antagonist function of Vpu by disrupting these two important interactions. To this end, we first established the BRET assay to monitor Vpu/BST-2 interaction in live cells. In this assay, YFP and luciferase (RLuc) were fused with Vpu and BST-2, respectively. A significantly higher than background BRET value was detected when RLuc-BST-2 and YFP-Vpu were co-expressed in HEK293T cells, which indicates the association of Vpu and BST-2 (Fig. 3A). This BRET value did not change when cells were treated with IMB-LA, suggesting that IMB-LA has no effect on the interaction of BST-2 and Vpu. To validate the BRET data, we further assessed the interaction between BST-2 and Vpu through co-immunoprecipitation. Vpu and BST-2-HA were co-expressed in HEK293T cells. BST-2-HA was immunoprecipitated from cellular lysates using anti-HA antibodies, and the presence of Vpu in the immunoprecipitates was successfully detected by western blotting (Fig. 3B). Again, IMB-LA failed to dissociate this interaction (Fig. 3B). We then performed BRET and co-immunoprecipitation experiments to assess the possible effect of IMB-LA on the interaction of β-TrCP2 and Vpu. As a control, we included the VpuS52A mutant that is unable to bind to β-TrCP2 and therefore generated only a very low BRET value in the presence of hRLuc-β-TrCP2 (Fig. 3C). Treatment with IMB-LA did not change the BRET value of hYFP-Vpu and hRLuc-β-TrCP2 (Fig. 3C). Similarly, no effect from IMB-LA was observed on the co-immunoprecipitation of β-TrCP2 by Vpu (Fig. 3D). Taken together, these data suggest that IMB-LA does not affect the interactions of Vpu with BST-2 or β-TrCP2.

### IMB-LA had no effect on the ubiquitination of BST-2

**Figure 4:**
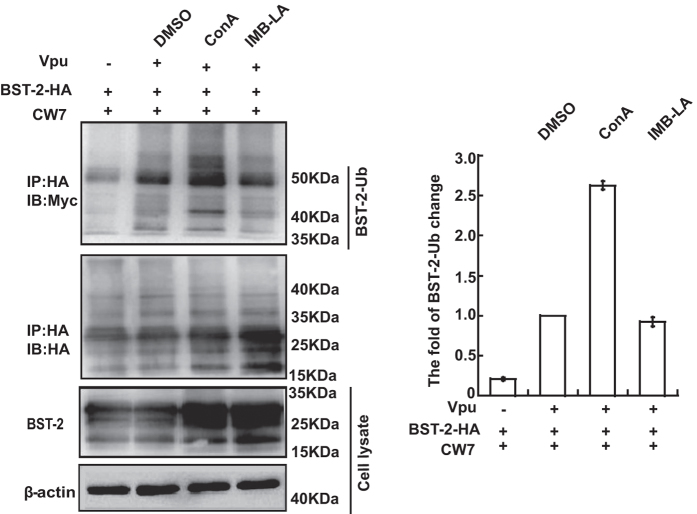
Effect of IMB-LA on the Vpu-induced ubiquitination of BST-2. HEK293T cells were co-transfected with pcDNA3.1-BST-2-HA, pcDNA3.1-Vpu and pCW7 (1:2:4). 24 h post-transfection, cells were treated with DMSO, 5 μM IMB-LA and 50 nM ConA for 24 h. Lysates were incubated with rabbit anti-HA antibody and then immunoprecipitated with protein A-agrose. The pre-IP lysates represented 1% of the IP input. The immunoprecipitated samples were analyzed by Western blot. The amount of ubiquitinated BST-2 was quantified using Image Lab^TM^ 2.0 software, and normalized with that of the group expressing BST-2 and Vpu in the presence of DMSO. Full-length blots/gels are presented in Supplementary data.

Studies have shown that Vpu causes ubiquitination of BST-2, which serves as a key signal for BST-2 degradation by the lysosomal machinery^41^. We therefore investigated whether IMB-LA affected Vpu-mediated BST-2 ubiquitination. First, BST-2-HA, Vpu, and Myc-tagged ubiquitin were transiently expressed in HEK293T cells, followed by the treatment of IMB-LA. BST2-HA was then immunoprecipitated from cellular lysates using anti-HA antibodies and the immunoprecipitates were analyzed by Western blotting. Ubiquitinated BST-2 was approximately 55 kDa, representing polyubiquitinated BST-2 as reported previously 41. Vpu stimulated ubiquitination of BST-2 by 2 to 3 folds. ConA treatment increased the amount of ubiquitinated BST-2, whereas IMB-LA exerted marginal effect on ubiquitination of BST-2 in the presence of Vpu (Fig. 4). These data suggest that IMB-LA has no effect on Vpu-induced ubiquitination of BST-2.

### IMB-LA blocks sorting of BST-2 into lysosomes in the presence of Vpu

**Figure 5:**
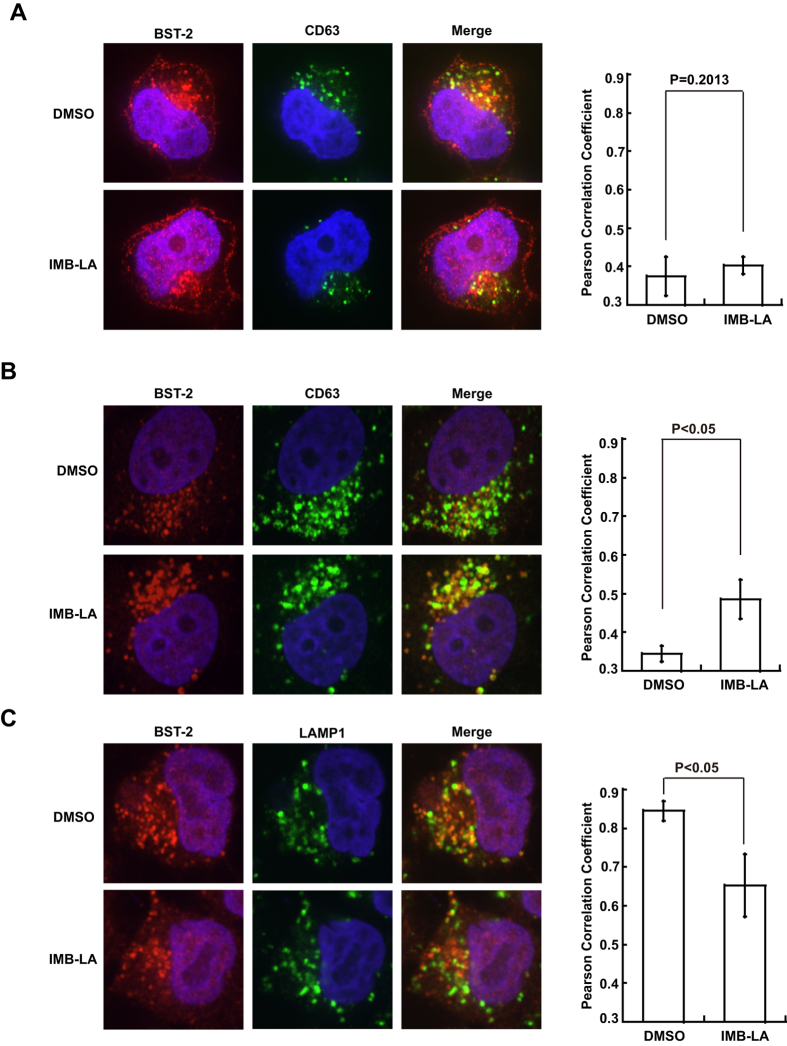
Effect of IMB-LA on cellular distribution of BST-2 in HeLa-Vpu cells. HeLa (**A**) and HeLa-Vpu cells (**B**) were treated with DMSO or 5 μM IMB-LA. 24 h later, cells were incubated with rabbit anti-BST-2 and mouse anti-CD63 antibodies. Cells were then stained by fluor-conjugated secondary antibodies. Images were recorded using a spinning disc confocal microscope. Signals of BST-2 are pseudocolored as red, CD63 as green. (**C**) HeLa-Vpu cells were incubated with rabbit anti-BST-2 and mouse anti-LAMP1 antibodies. BST-2 signals are shown in red, LAMP1 in green. The left panels in (**A**,**B**) and (**C**) graphically represent the results of the Peason’s Correlation coefficients of the two channels in each set (Fig. 5, left panel).

To be degraded in lysosomes, the ubiquitinated BST-2 needs to be sorted into lysosomal compartments. Indeed, in HeLa-Vpu cells that stably express Vpu, strong colocalization of BST-2 with the lysosomal marker LAMP1 was observed (Fig. 5). This observation is based on the analysis of ten views of each set of micrographs that were randomly selected and the results of the Pearson’s Correlation Coefficients of the two channels in each set (Fig. 5, left panel). IMB-LA treatment led to a striking increase in the co-localization of BST-2 with the late endosomal marker CD63 in HeLa-Vpu cells (Fig. 5B), whereas no effect was observed in HeLa cells that do not contain Vpu (Fig. 5A). Concomitant with this increase, colocalization of BST-2 with LAMP1 dramatically diminished in HeLa-Vpu cells (Fig. 5C), which suggest that IMB-LA blocks the sorting of BST-2 into lysosomes for degradation in the HeLa-Vpu cells.

### IMB-LA inhibits HIV-1 replication in a BST-2-dependent manner

**Figure 6:**
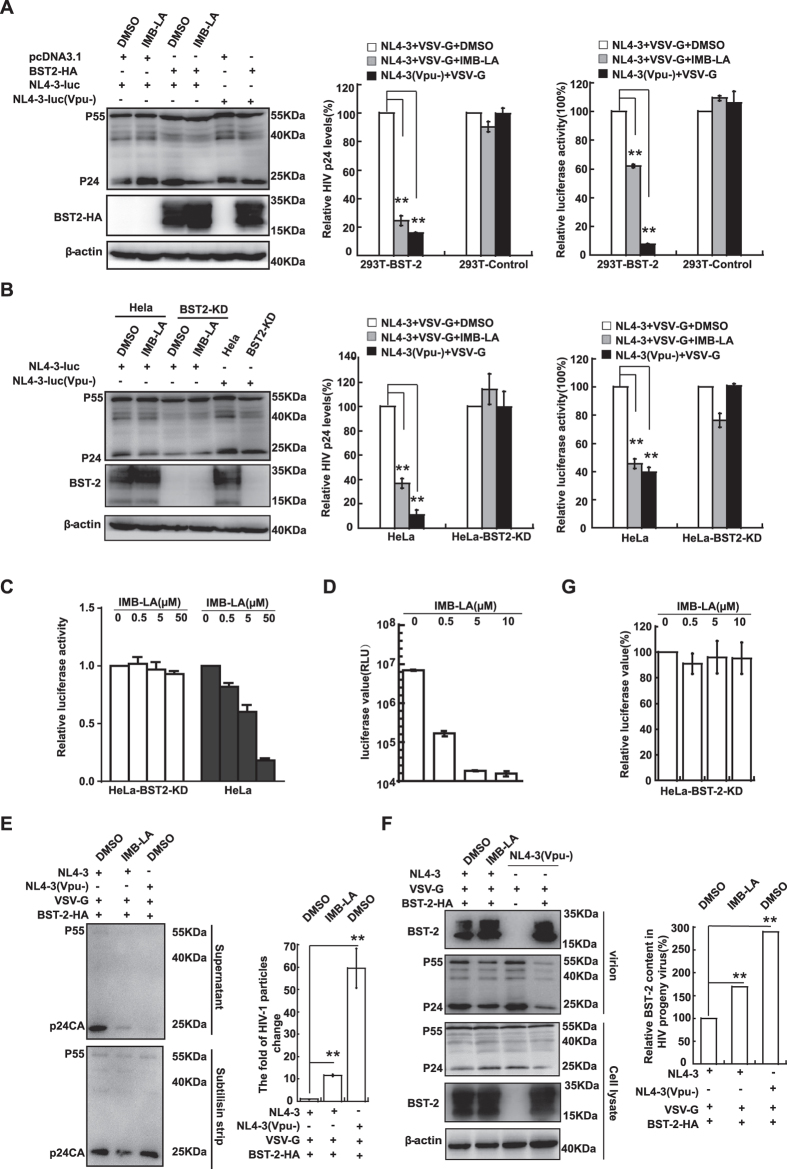
IMB-LA inhibits HIV-1 infection in BST-2-expressing cells. (**A**) 293 T cells and 293T-BST-2 were co-transfected with 300 ng pNL4-3luc(R-E-), 210 ng pHIT/G and 200 ng pcDNA3.1-BST-2-HA. The Vpu-negative pNL4-3luc (−Vpu) was used as a control. 24 h post-transfection, cells were treated with DMSO or 5 μM IMB-LA for 24 h. Cellular content of Gag and BST-2-HA were analyzed by Western blot using anti-p24 antibody and anti-HA antibody, respectively. β-actin was detected as a control (left panel). Amounts of viruses produced from the transfected cells were determined by p24CA ELISA (middle panel). The progeny viruses containing the same amount of p24 were used to infect SupT1 cells and HIV-1 infection was determined by measuring firefly luciferase activities (right panel). (**B**) HeLa and HeLa-BST-2-KD cells were transfected with 500 ng pNL4-3luc(R-E-) or pNL4-3luc (-Vpu) and 350ng pHIT/G. Protein expression in cells (left panel), amounts of viruses (middle panel) and infectivity of the progeny viruses (right panel) were determined as described in Fig. 6A. (**C**) 5 × 10^5^ HeLa or HeLa-BST2-KD cells were transfected with 0.3μg NL4-3deltaLuc and 0.3 μg Env, and then were treated with DMSO or IMB-LA, respectively. The supernatant was used to infect TZM-bl cells and luciferase activity was measured. (**D**) TZM-bl cells were infected with wild type NL4-3 viruses and then incubated with the indicated compounds, followed by luciferase activity measurement. (**E**) 293T cells were transfected with pcDNA3.1-BST-2 and either HIV-1(WT) or HIV-1(delVpu), followed by treatment with either DMSO or 5 μM IMB-LA. Released particles were harvested from culture supernatants before (top panel) and after (bottom panel) subtilisin strip, respectively, and then subjected to Western blot analysis. (**F**) HEK293T cells were co-transfected with pcDNA3.1-BST-2-HA, pNL4-3luc(R-E-) and pHIT/G as described in Figure 6 (**A**). Cells and virions were subjected to western blot analysis. (anti-p24 and anti-HA, as indicated). (**G**) The HeLa-BST-2-KD cells were exposed to virion supernatants containing the same amount of p24. 24 h later, cells were treated with IMB-LA for 24 h. Firefly luciferase activities in cell lysate were determined. Each bar represents the mean and standard deviation of at least three independent experiments. **denotes *P* < 0.01. Full-length blots/gels are presented in Supplementary data.

We next investigated whether IMB-LA was able to inhibit HIV-1 release and infectivity in the presence of endogenous or exogenous BST-2 through its ability of blocking Vpu-mediated BST-2 degradation. The experiments were carried out in HeLa and HEK293T cells. HeLa cells express endogenous BST-2 whereas HEK293T cells have no detectable BST-2 expression. We generated one cell lines, HeLa-BST2-KD that was depleted of endogenous BST-2 with shRNA. As for HEK293T cells, we overexpressed pcDNA3.1-BST-2-HA (named 293T-BST-2) and pcDNA3.1 (named 293T-control) for exogenous BST-2 test. We co-transfected these cell lines with pNL4-3Luc(R-E-) and pHIT/G to produce VSV-G pseudotyped viruses with or without IMB-LA treatment. Western blot analysis of virus-producing cells showed that IMB-LA increased the level of BST-2 compared with that of DMSO-treated 293T -BST-2 and HeLa cells, and exhibited no effect on Gag expression (Fig. 6A and B, left panel). In addition, production of progeny virions was determined by measuring HIV-1 p24CA with ELISA. BST-2 significantly diminished the release of Vpu (−) but not the Vpu ( + ) HIV-1 (Fig. 6A and B, middle panel). IMB-LA did not affect HIV-1 production in the HEK293T cells and the HeLa-BST2-KD cells, both of which did not express BST-2. In contrast, IMB-LA reduced HIV-1 production by 80% and 60% in 293T-BST-2 and HeLa cells both of which expressed BST-2 respectively (Fig. 6A and B, middle panel). In addition to the reduction in viral particle releasing, released viruses derived from BST-2 containing cells exhibited impaired infectivity (Fig. 6A and B, right panel). Furthermore, IMB-LA showed a similar inhibitory effect on the infectivity of virions with HIV-1 Env protein instead of VSV-G (Fig. 6C). These data indicate that IMB-LA exerts its inhibition on HIV-1 production and infectivity mainly dependent of BST-2 expression.

We next asked whether IMB-LA is capable of inhibiting the replication of wild type HIV-1 in the presence of BST-2. We therefore investigated the effect of IMB-LA on HIV-1 replication in TZM-bl cells, which are derived from HeLa and express endogenous BST-2. The results showed that IMB-LA inhibited HIV-1 replication by 2 to 3 orders of magnitude at concentrations from 2.5–10 μM (Fig. 6D), which demonstrates a potent anti-HIV activity of IMB-LA. Moreover, we performed a subtilisin strip assay that has been widely used to analyze BST-2 mediated tethering effect on virion releasing. The result showed that subtilisin treatment, which is able to cleave BST-2 and thereby releasing virions tethered at cell surface by BST-2, significantly increased the amount of virions in the supernatant from 293T cells treated with IMB-LA (Fig. 6E). This provides further evidence supporting that IMB-LA treatment caused the accumulation of virions tethered by BST-2 at cell surface, resulting in the inhibition on HIV-1 replication.

We have previously shown and presented herein that, in addition to inhibit virus release, BST-2 also diminishes HIV-1 infectivity 15. In addition to the reduction in viral particle releasing, released viruses derived from BST-2 containing cells exhibited impaired infectivity (Figs 6A and B, right panel). We therefore asked whether IMB-LA also causes the incorporation of BST-2 into HIV-1 particles and consequently decreases virus infectivity. Indeed, the level of BST-2 in HIV-1 particles increased by 70% when 293T-BST-2 virus producer cells were treated by IMB-LA (Fig. 6F). When these virus particles were used to infect SupT1 cells, their infectivity dropped by 40%–60%, whereas no effect from IMB-LA was detected on the infectivity of viruses that were produced from HeLa-BST2-KD and HEK293T cells that do not express BST-2 (Fig. 6A and B). Taken together, these data suggest that IMB-LA inhibits HIV-1 in a BST-2-dependent manner and that this antiviral effect is a result of two actions, i.e., reducing the release of virus particles and diminishing virus infectivity. In agreement with it, a marginal effect (approximate 10%) was observed on the infectivity when IMB-LA (up to 5 μM) was added either during or prior to infection. This suggests that IMB-LA exhibit some non-specific but marginal effect on HIV-1 infectivity (Fig. 6G).

## Discussion

For the first time, we have identified a small molecule compound IMB-LA that inhibits HIV-1 infection by exposing HIV-1 to BST-2 restriction. IMB-LA /Lapachol is a naphthoquinone [2-hydroxy-3-(3-methyl-2-butenyl-)-1,4-naphthoquinone] which has been reported to possess multiple therapeutical effects including anti-malarial and anti-cancer activities^42^,^43^. However, its antiviral activities have not been reported up to date. IMB-LA does so by specifically preventing Vpu-mediated down-regulation of BST-2 and thereby restoring BST-2 expression at the cell surface (Figs 1 and 2). Further studies showed that IMB-LA diminished localization of BST-2 to lysosomes and thereby rescuing BST-2 from lysosomal degradation (Fig. 5). IMB-LA does not affect the interaction of Vpu with BST-2 or β-TrCP2 (Fig. 3), nor modulates the ubiquitination of BST-2 (Fig. 4). It is possible that IMB-LA blocks the transfer of ubiquitinated BST-2 into lysosomes and thereby inhibits BST-2 degradation. Most importantly, HIV-1 release and infection are markedly inhibited by IMB-LA in BST-2 expressing cells. This study provides a proof-of-concept that utilizes small molecule compounds to block viral antagonism and expose viruses to innate immune response.

One major concern in drug development is the specificity of the compounds toward the target. In the context of IMB-LA, one issue is whether this compound exerts a general effect on the endosomal/lysosomal pathway by which Vpu degrades BST-2, especially given that the H + ATPase inhibitor ConA is able to prevent Vpu from down-regulating BST-2 by blocking the lysosomal pathway. This possibility was refuted by the results showing that IMB-LA did not affect the degradation of IFNAR1 in response to IFNα (Fig. 2). IFNAR1 is recognized by β-TrCP2 that recruits E3 ligase to IFNAR1 through binding to the phosphor-motifs DpSGXXpS in its substrate. Therefore, IMB-LA does not likely exert a general impact on lysosome-dependent protein degradation.

In addition to antagonizing BST-2, Vpu has another important function to down-regulate CD4, the receptor of HIV-1. This function provides another opportunity to test the specificity of IMB-LA in targeting Vpu/BST-2. No effect of IMB-LA on Vpu-mediated down-regulation of CD4 was observed (Fig. 2). Since Vpu utilizes the β-TrCP2-containing ubiquitin E3 ligase to degrade both CD4 and BST-2, it can be concluded that IMB-LA has a great selectivity to target Vpu/BST-2 pathway. In support of this conclusion, results of both BRET and co-immunoprecipitation experiments showed no effect of IMB-LA on the interaction of Vpu with β-TrCP2 and BST-2.

Enveloped viruses have developed different means to counter BST-2. For example, the K5 protein of KSHV is an ubiquitin E3 ligase and ubiquitinates the K18 residue at the cytoplasmic region of BST-2. This ubiquitination event leads to localization of BST-2 to lysosomes and subsequent degradation. The observation that IMB-LA did not affect K5-mediated down-regulation of BST-2 further supports the high specificity of this compound toward targeting the antagonist function of Vpu.

How does IMB-LA block Vpu from antagonizing BST-2 ? We pursued this question on the basis of the known mechanisms of Vpu antagonism. First, Vpu needs to interact with BST-2 through their transmembrane domains^26^,^44^. A number of key residues across their transmembrane domains have been identified that are essential for this interaction and their mutations abrogate Vpu counteraction of BST-2^30^,^31^. Our data of both BRET and co-immunoprecipitation experiments showed no effect of IMB-LA on Vpu and BST-2 interaction, suggesting that IMB-LA does not target the transmembrane domains of these two proteins. Second, Vpu utilizes its S52/S56 motif to recruit β-TrCP2^45^. This interaction is essential for BST-2 polyubiquitination and subsequent degradation in lysosomes^17^,^18^. IMB-LA does not affect this interaction either. In addition, no effect of IMB-LA was seen on the degree of BST-2 ubiquitination. Third, the ubiquitinated BST-2 is sorted to lysosomes for degradation^46^. In the presence of IMB-LA, localization of BST-2 to lysosomes is drastically decreased with concomitant increase of BST-2 in CD63 + endosomal compartments. This observation suggests that IMB-LA specifically blocks the sorting of BST-2 into lysosomes and thus inhibits Vpu-induced degradation of BST-2. Subsequently, the inhibition of BST-2 degradation may restore BST-2 level at cell surface as conA does.

We currently do not know how IMB-LA prevents the sorting of ubiquitinated BST-2 into lysosomes. It is known that not all the components in late endosomes are destined for degradation in lysosomes^47^,^48^. For example, some endosomes can fuse with plasma membranes and release as exosomes into the extracellular environment. Late endosomes can also backfuse with limiting membranes which leads to the release of toxin or RNA into the cytoplasm in a process that is controlled by LBPA, ALIX and ESCRTs^47^. LDL-derived cholesterol can utilize this back-fusion process to escape from sorting into lysosomes^47^. It is known that Ub-cargos are sorted by receptors that recognize the ubiquitin chains^49^. These Ub receptors include the Hrs/STAM complex, CGA proteins and Tom1-Tollip complex^48^,^50^. Loss of CGA and Tom1-Tollip results in sorting defect of particular Ub-cargoes^50^,^51^. One study reported that HRS is required for BST-2 down-regulation by Vpu^52^. It would be interesting to test whether IMB-LA affects this function of HRS.

Although it is not completely clear how IMB-LA blocks BST-2 degradation, IMB-LA inhibits HIV-1 infection only in cells that express BST-2. IMB-LA, in the presence of Vpu, allows BST-2 to express at the cell surface and enables BST-2 to block HIV-1 release and also to diminish the infectivity of nascent virus particles. Given the great effectiveness and importance of interferon in protecting the hosts from infection by a vast diversity of viruses, our study further supports the feasibility of utilizing small molecule compounds to disarm viral antagonism and expose viruses to the restriction of innate immune responses, especially for persistent viral infection^53^. This new generation of antiviral strategy also has a utility as vaccine adjuvant to boost local innate immunity.

## Methods

### Plasmids, antibodies and compounds

The plasimid pVphu expresses codon-optimized Vpu. The vesicular stomatitis virus glycoprotein (VSV-G) expressing vector pHIT/G and HIV-1 proviral indicator construct pNL4-3Luc(R-E-) were provided by Johnny He. pNL-Luc.R-E-Vpu- has the first ATG of Vpu coding sequence changed to ACG and expresses luciferase as the reporter. pcDNA-IFNAR1-FLAG and pcDNA-β-TrCP2-HA were provided by Serge Y. Fuchs. pcDNA3.1-BST2-HA was from Paul D. Bieniasz. pcDNA3.1-K5-HA was from Klaus Früh. pCW7 that expresses Myc-tagged ubiquitin was provided by Xiaofang Yu. Anti-Vpu rabbit antibody and anti-BST-2 rabbit antibody were obtained from the National Institutes of Health (NIH) AIDS Research & Reference Reagent Program. Mouse anti-BST-2 antibody, mouse anti-CD63 antibody and mouse anti-LAMP1 antibody were purchased from Abcam. HA antibody, beta-actin antibody, HRP (horseradish peroxidase)-labeled donkey anti-rabbit, HRP-labeled rabbit anti-mouse and goat anti-rabbit IgG-FITC secondary antibodies were purchased from Santa Cruz Co. Anti-mouse IgG-FITC secondary antibody and anti-rabbit IgG-TRITC secondary antibody were purchased from Zhong Shan Gold Bridge. Concanamycin A, MG132 and IMB-LA/ Lapachol were purchased from Sigma. IMB-LA was resolved in DMSO (solubility > 242.27mg/ml).

### Cells and transfections

HeLa, HEK293T, HeLa-Vpu, HeLa-BST2-KD and TZM-bl cells were cultured in DMEM (GBICO) supplemented with 10% fetal bovine serum (FBS) (GIBCO). SupT1 cells were maintained in RPMI-1640 (Hyclone) containing 10% FBS. HeLa-Vpu cells were generated by transfection of pVphu vectors and then selected with 400μg/ml G418. HeLa-BST2-KD cells were generated by transfection of shRNA-BST2 and puromycin (500ng/ml) selection. HeLa cells were transfected using Fugene HD transfection reagents (Roche), according to the manufacturer’s instruction. Transfections of HEK293T were performed using Lipofectamine 2000 (Invitrogen) according to the manual.

### Cell-ELISA

The Cell-ELISA was carried out as described previously^34^. In brief, HeLa-Vpu cells were seeded into 96-well plates. Twenty-four hours later, cells were treated with drugs for 24h. Cells were then washed with PBS at room temperature, followed by the fixation with 4% paraformaldehyde (PFA) (100μl/well). After fixation for 20 minutes, cells were incubated with BST-2 antiserum for 1h at 37 °C and then washed with PBS. HRP-labeled second antibody (1:5000) was added to each well. After incubation for 40 min at room temperature, 100 μl TMB substrate solution was added and incubated for another 30 min at room temperature avoiding light. Finally, the reaction was stopped by adding 50μl of 0.5M HCl. The absorption at 450nm was measured immediately.

### Flow cytometry

For analysis of cell surface BST-2, HeLa-Vpu cells were treated with IMB-LA or DMSO for 24h. Then the cells were trypsinized and resuspended in flow cytometry buffer (containing 10% fetal bovine serum), and following incubated with anti-BST-2 rabbit antibody in 2% BSA at 4 °C for 1h. The cells were then washed with prechilled PBS containing 2% BSA for 4 times followed by staining with anti-rabbit IgG conjugated with FITC for 30 min, then analyzed on a FACS caliber system (BD biosciences).

### Virion release and infectivity analysis

To assess the effect of IMB-LA on the release of progeny virions, the experiments were carried out in HEK293T and HeLa cells. HEK293T cells were co-transfected with 2μg pNL4-3Luc(R-E-), 1.4μg pHIT/G and 300ng BST-2-HA (or pcDNA3.1) in 10-cm dishes. HeLa and HeLa-BST2-KD cells were co-transfected with 3μg pNL4-3Luc(R-E-), 2.1 μg pHIT/G. After 20 h, the media was changed and IMB-LA was added. 24h later, the cells were harvest for western blotting, and the supernatants were collected and filtered through a 0.45 μm filter. The amounts of viruses were determined by measuring HIV-1 p24CA antigen with antigen enzyme-linked immunosorbent assay (p24CA ELISA) (Vironostika HIV-1 Antigen) and ultracentrifugation assay. The p24CA ELISA assay was peformed according to the manufacturer’s instruction. For ultracentrifugation, the supernantants were pelleted through a 20% sucrose cushion at 35000 rpm for 60 min, and harvest the viral sample for western blotting.

To assess the infectivity of HIV-1, the SupT1 cells (1 × 10^5^) in 96-well plates were exposed to virion supernatants containing the same p24 value. Forty-eight hours later, SupT1 cells were lysed and firefly luciferase activities were determined using a firefly Luciferase Assay System (Promega).

To assess the effect of IMB-LA on the replication of wild type HIV-1, 2 × 104 TZM-bl cells were infected with WT NL4-3 viruses at MOI = 0.1 for 8h. 8h post-infection, the medium was refreshed and the cells were incubated with the indicated compounds above for another 72 h. Cells were harvested and luciferase activity was measured.

### Co-Immunoprecipitation

For BST-2 and Vpu IP assay, HEK293 T cells were seeded in 10cm dishes. 24 h later, the cells were cotransfected with 2 μg Vpu and 2μg BST-2-HA. Another 24 h later, the cells were treated with DMSO or 5μM IMB-LA for 24 h. At last, the cells were collected and lysated in NP-40-containing buffer for 30min on ice. Cell lysates were centrifugated at 10,000 × g for 10 min at 4 °C and supernatant was transferred to a fresh 1.5 ml tube on ice. The cell lysates were incubated with anti-HA rabbit antibody for 3h at 4 °C. 30μl of proteinA-Agarose (Santa Cruz) was added and incubated overnight at 4 °C. The beads were washed 4 times with lysis buffer (cold) and at last boiled in 40 μl sample buffer for 5–10 min. The samples were analyzed by Western blotting.

For Vpu and β-TrCP2-HA IP assay, HEK293T cells were seeded in 10cm dishes. 24 h later, the cells were cotransfected with 2 μg Vpu, 2 μg BST-2-FLAG and 2 μg TrCP2-HA. The following experimental procedures were the same as Vpu and BST-2 IP assay.

### Ubiquitination Assay

Ubiquitination assay was performed as previously reported^41^. In brief, 70–80% confluent HEK293T cells were transfected with 2.5 μg BST-2-HA, 5μg of Vpu, and 10 μg pCW7-Ubquitin-Myc in 10-cm dishes. Twenty-four hours later, IMB-LA was added. Another 24 h later, the cells were collected and lysed in NP-40-containing buffer for 30 min on ice. Cell lysates were centrifugated at 10,000 × g for 10 min at 4 °C and supernatant was transferred to a fresh 1.5 ml tube on ice. The cell lysates were incubated with anti-HA rabbit antibody for 3h at 4 °C. 30 μl of proteinA-Agarose (Santa Cruz) was added and incubated overnight at 4 °C. The beads were washed 4 times with cold lysis buffer and then boiled in 40 μl sample buffer for 5–10 min. The samples were analyzed by Western blotting.

### Confocal microscopy

HeLa-Vpu (or HeLa) cells were treated with IMB-LA for 24 h, then washed with cold PBS, fixed with 4% PFA for 15 min and permeabilized with 0.2% Triton X-100 for 10 min. Cells were incubated with anti-BST-2 and anti-CD63 antibodies (or anti-LAMP1 antibodies) overnight at 4 °C, followed by washing with PBS, and further incubated with the secondary antibodies for 1 h at room temperature. Images were recorded by an UltraVIEW vox spinning disc confocal scanning system (Perkin Elmer) on a Olympus IX 81 microscope.

Pearson’s Correlation Coefficients was used to assess the colocalization of two protein obseved in confocal microscope. This index is a measure of the strength and direction of the linear relationship between two variables that is defined as the (sample) covariance of the variables divided by the product of their (sample) standard deviations. Formula is shown was followed


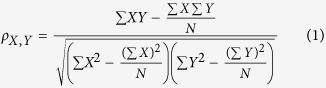


### Real time PCR

Total RNA was extracted from cells using TRIZOL Reagent (Invitrogen) following the instruction. RNA was converted to cDNA using M-MLV Reverse Transcriptase (Promega) with random primers. The cDNAs were quantified using SsoFast EvaGreen Supermix (Bio-Rad) and Bio-Rad iCycler iQ5 Real-Time PCR systems. Primer sequences for quantifying cDNAs were as follows: BST-2 (sense: CTGCAACCACACTGTGATG, antisense: ACGCGTCCTGAAGCTTATG)^19^, GAPDH (sense: GTCCACTGGCGTCTTCACCA, antisense: GTGGCAGTGATGG CATGGAC). Level of GAPDH mRNA was used as an internal control to normalize the level of BST-2 mRNA.

### BRET assay

The BRET assay was used to monitor protein-protein interaction as previously described^54^. Briefly, HEK293T cells were co-transfected with hYFP-Vpu and hRLuc-BST2 (or β-TrCP2-RLuc), and harvested 48h after transfection. Cells were then distributed into 96-well microplates (white Optiplate; Perkin-Elmer) with a density of 1 × 10^5 ^cells/well. Rluc substrate coelenterazine h was added at a final concentration of 5 μM. The signals were detected at 480 (±10) nm and 535 (±10) nm. BRET ratio was determined as “emission at 535 nm/ emission at 475 nm”.

### Subtilisin Strip Assay

For Subtilisin Strip Assay, 293 T cells were transfected with pcDNA3.1-BST-2, HIV-1(WT) or HIV-1(delVpu). 24 h later, the cells were treated with DMSO and 5 μM IMB-LA for another 24h. Constitutively released particles were harvested from culture supernatants. Thereafter, cells were incubated for 15 min at 37 °C in buffer (10mM of Tris/HCl (pH 8.0), 150 mM NaCl, 5 mM CaCl2) with or without the addition of subtilisin (100 μg/ml). The stripping reaction was stopped with DMEM/FBS containing 5 mM PMSF. Cells were pelleted and the supernatants were filtered (0.2 mm) before harvesting the released virion particles, by pelleting through sucrose.

### Statistical analysis

All statistical analyses were carried out using the KaleidaGraph 4.0 statistical software. Values and error bars in the bar graphs represent the means and standard deviations of results from at least three independently performed experiments. Comparisons between groups for statistical significance were carried out with the 2-tailed paired Student’s t-test. In all cases, P < 0.05 was considered statistically significant.

### Drug Screening

A high-throughput screen (HTS) Cell-ELISA assay was exploited to perform a primary screen against a library of 56000 compounds at a concentration of 10 μM, which include Microsource Spectrum Collection (2000), NCI Diversity Set I (1974), NCI Diversity Set II (1364) and natural product collection at the Institute of Medicinal Biotechnology. A positive hit was defined as a compound that increases cell ELISA optical density at OD450 by more than 3 standard deviations (SD). 75 positive hits from the primary screen were further tested for cellular toxicity effects, dose-response assessments and anti-HIV activities.

## Additional Information

**How to cite this article**: Mi, Z. *et al.* A small molecule compound IMB-LA inhibits HIV-1 infection by preventing viral Vpu from antagonizing the host restriction factor BST-2. *Sci. Rep.* **5**, 18499; doi: 10.1038/srep18499 (2015).

## Supplementary Material

Supplementary Information
