# Supplementary material for: A small molecule compound IMB-LA inhibits HIV-1 infection by preventing viral Vpu from antagonizing the host restriction factor BST-2
Source: Sci Rep. 2015 Dec 16;5:18499. doi: 10.1038/srep18499 (PMC4680884; doi:10.1038/srep18499)
Supplement: Supplementary Information [file srep18499-s1.pdf]

A small molecule compound IMB-LA inhibits HIV-1 infection by preventing viral Vpu from antagonizing the host restriction factor BST-2

Zeyun Mi[1,5]*, Jiwei Ding[1]*, Quan Zhang[1]*, Jianyuan Zhao[2], Ling Ma[1], Haisheng Yu[6], Zhenlong Liu[3], Guangzhi Shan[1], Xiaoyu Li[1], Jinming Zhou[1], Tao Wei[2], Liguo Zhang[6], Fei Guo[4], Chen Liang[3§], Shan Cen[1§]

[1] Institute of Medicinal Biotechnology, Chinese Academy of Medical Sciences and Peking Union Medical School, Beijing 100050, China;

[2] Beijing Union University, Beijing 100101, China

[3] Lady Davis Institute-Jewish General Hospital, McGill University, Montreal, Quebec, Canada;

[4] Institute of Pathogen Biology, Chinese Academy of Medical Science, Beijing, China.

[5] Department of Biochemistry and Molecular Biology, Tianjin Medical University, Tianjin, China

[6]Institute of Biophysics, Chinese Academy of Sciences, Beijing, China

*These authors contributed equally to this work

[§] Corresponding author: Shan Cen, Tel: 86-10-63037279; Fax: 86-10-63037279; E-mail: shancen@imb.pumc.edu.cn; or Chen Liang, Tel: 1-514-3408260; Fax: 1-514-3407537; E-mail: chen.liang@mcgill.ca

Running title: A compound inhibits Vif-mediated degradation of BST-2.

Key words: HIV-1, BST-2, Vpu, Tetherine, small molecule compound

Fig 1D

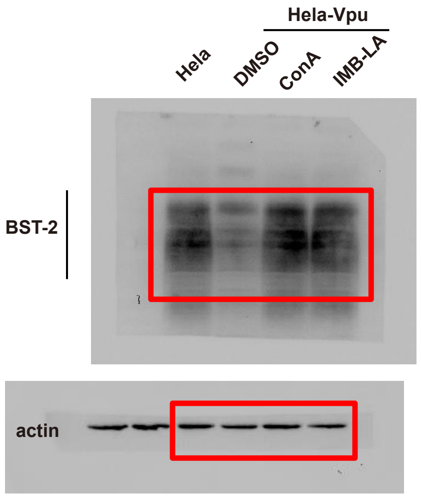

Fig 2B

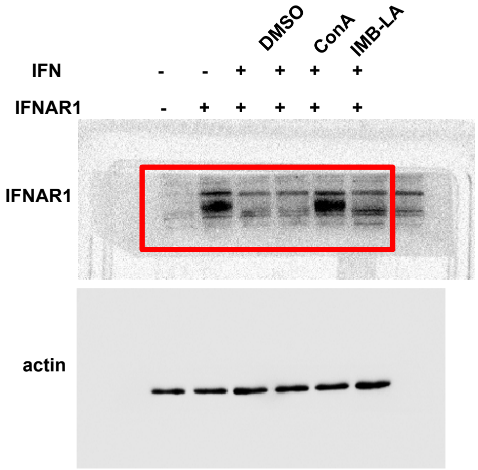

Fig 3B

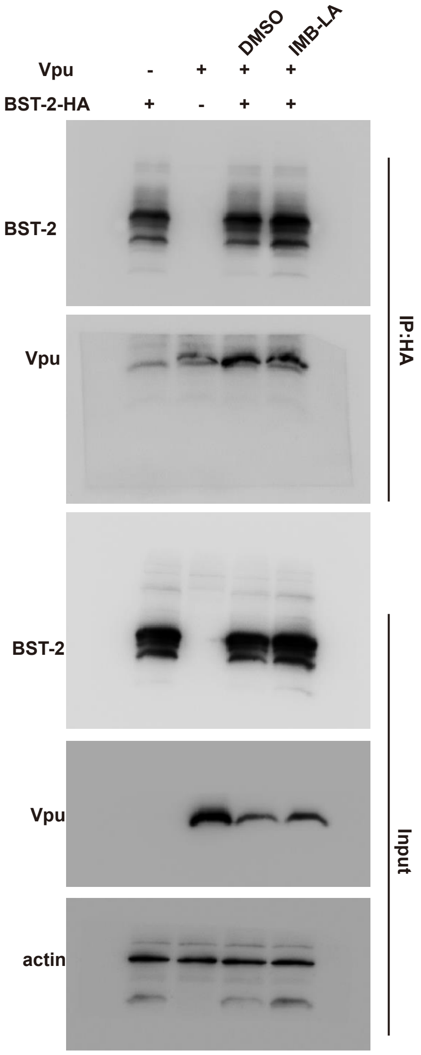

Fig 3D

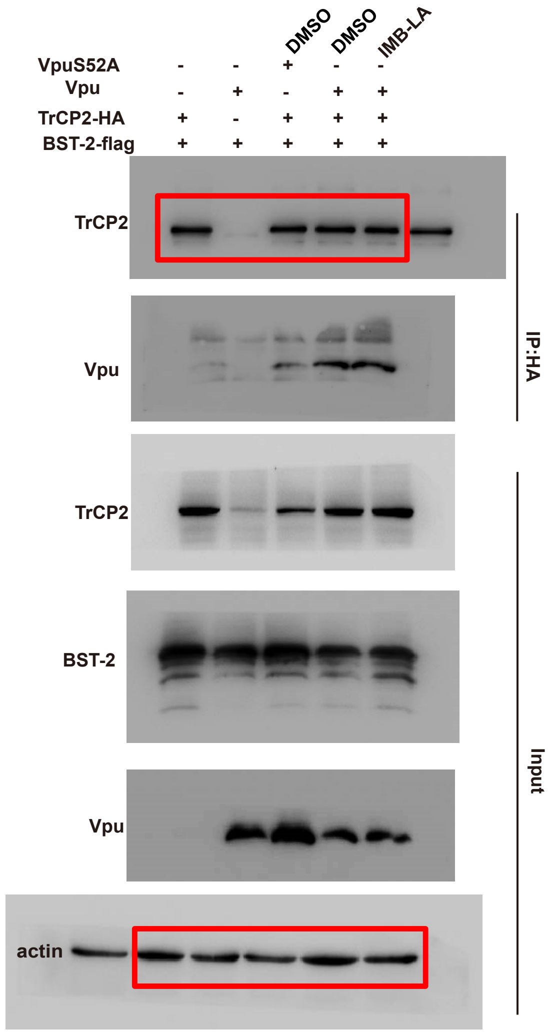

Fig 4

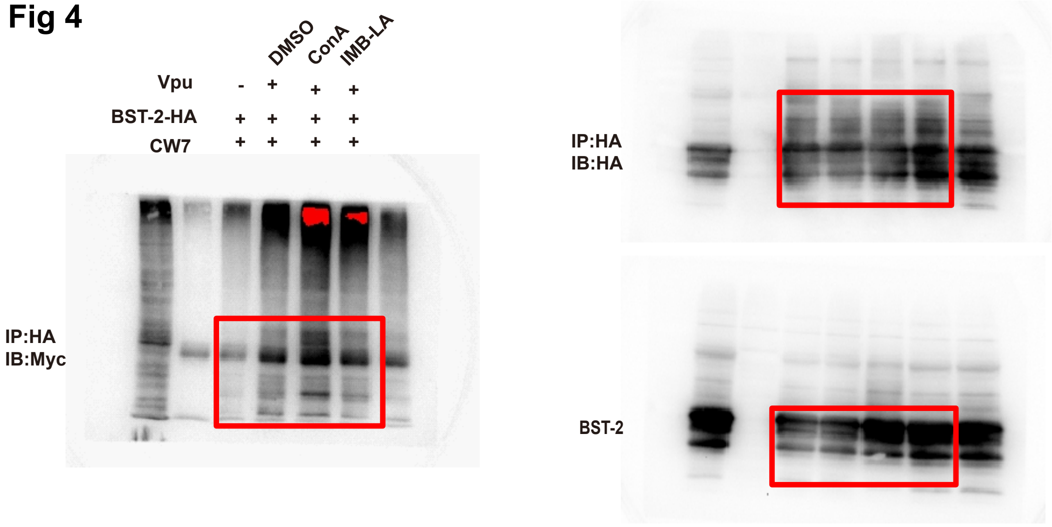

Fig 6A

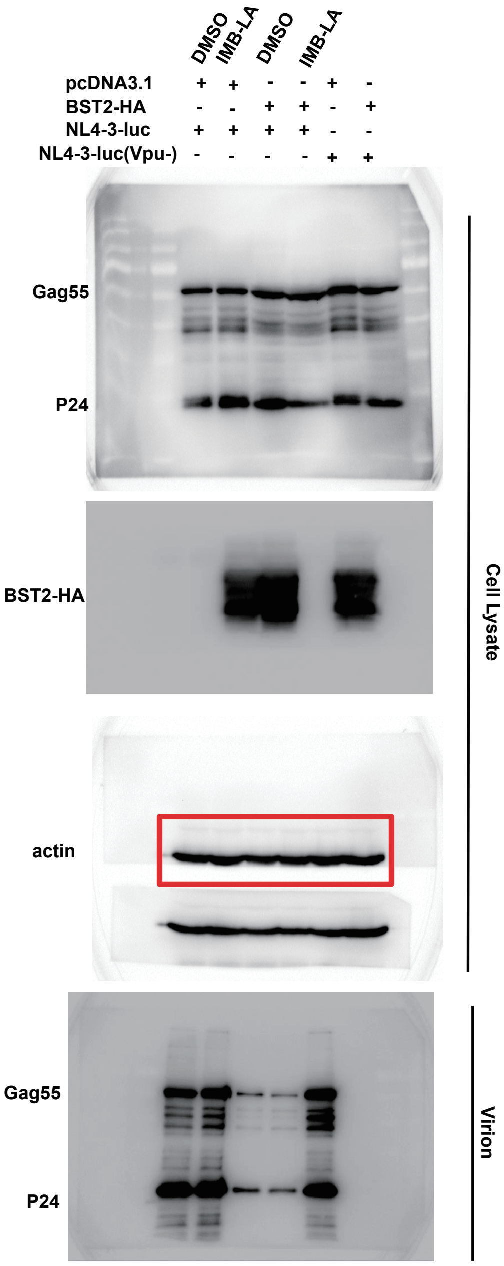
DMSO
IMB-LA
DMSO
IMB-LA
pcDNA3.1 + + - - + -
BST2-HA - - + + - +
NL4-3-luc + + + + - -
NL4-3-luc(Vpu-) - - - - + +
Gag55
P24
BST2-HA
actin
Cell Lysate
Gag55
P24
Virion

Fig 6B

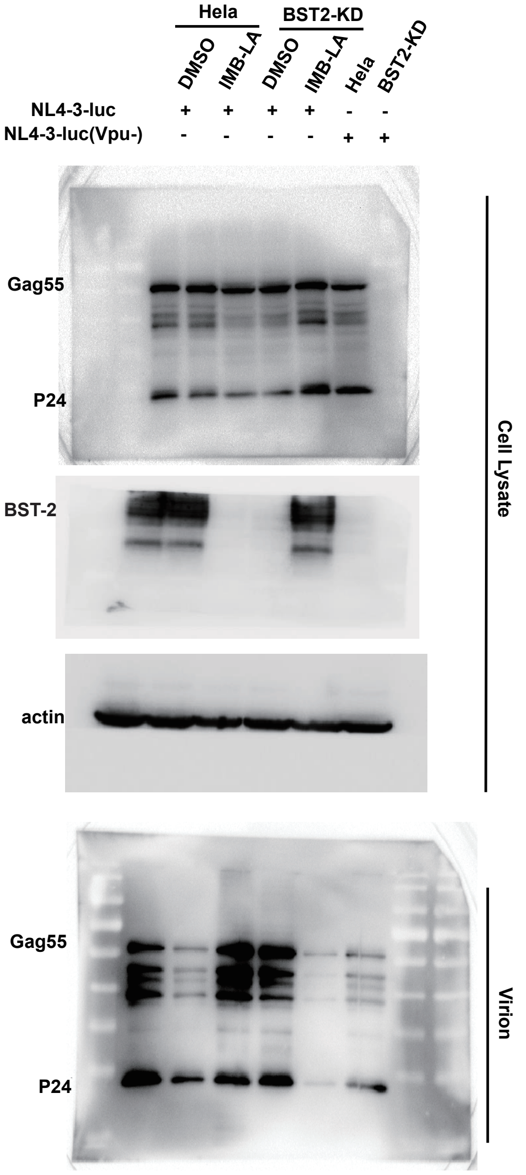
Hela
BST2-KD
DMSO
IMB-LA
DMSO
IMB-LA
Hela
BST2-KD
NL4-3-luc + + + + - -
NL4-3-luc(Vpu-) - - - - + +
Gag55
P24
BST-2
actin
Cell Lysate
Gag55
P24
Virion

FIG 6G

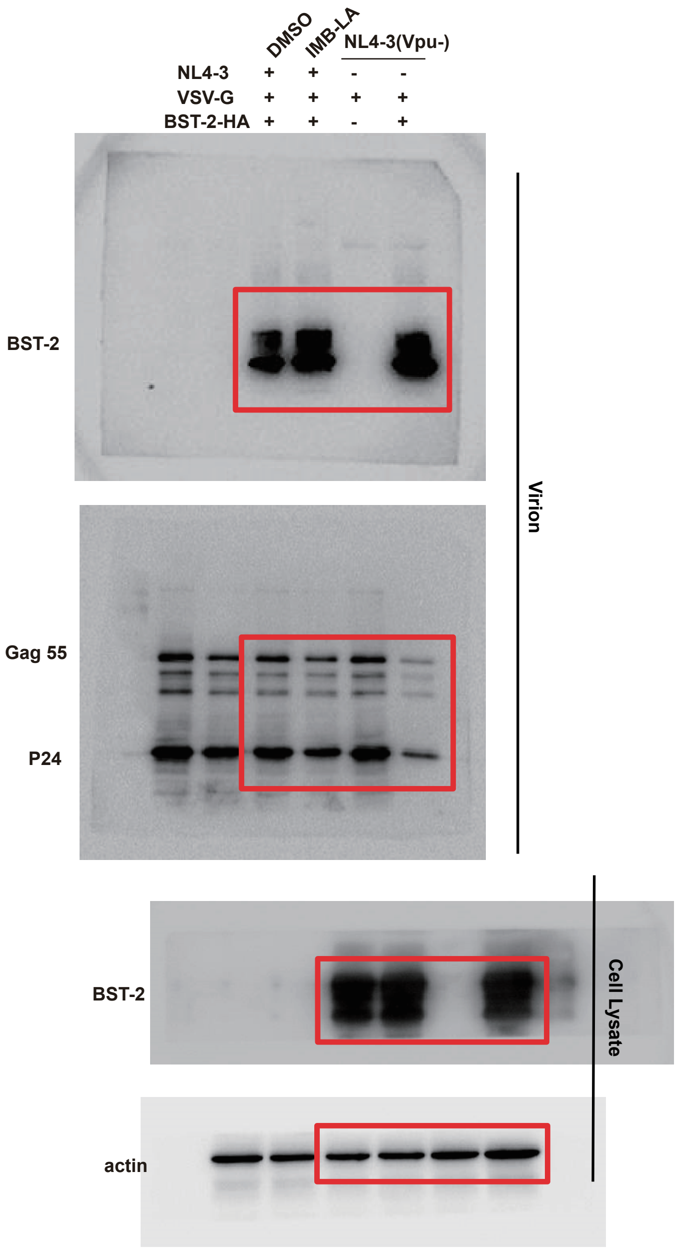
DMSO
IMB-LA
NL4-3(Vpu-)
NL4-3 + + - -
VSV-G + + + +
BST-2-HA + + - +
BST-2
Gag 55
P24
Virion
BST-2
Cell Lysate
actin
